# OCT Angiography–Based Quantitative Choroidal Vasculature Analysis in Choroidal Melanomas and Nevi

**DOI:** 10.1016/j.xops.2025.100979

**Published:** 2025-10-23

**Authors:** Tekla Kurdiani, Miltiadis Fiorentzis, Nikolaos E. Bechrakis, Tobias Kiefer

**Affiliations:** Department of Ophthalmology, University Hospital Essen, University Duisburg-Essen, Essen, Germany

**Keywords:** Choroidal melanoma, Choroidal nevus, Choroidal vascular analysis, OCT angiography

## Abstract

**Purpose:**

Differentiation between choroidal melanoma (CM) and choroidal nevus (CN) is primarily made clinically with the aid of multimodal imaging. Both entities can be distinguished by their vascular architecture in histopathology. OCT angiography (OCT-A) is a noninvasive method to visualize retinal and choroidal vasculature and has rarely been used to differentiate CM and CN, and if so, exclusively descriptively. The purpose of this study was to differentiate both entities via OCT-A using an open-source vascular analysis software (OCTAVA), using quantitative choroidal vasculature analysis.

**Design:**

Retrospective case-control study.

**Subjects:**

Treatment-naïve patients with CM and CN who obtained OCT-A of the tumor at the University Hospital Essen between November 2022 and December 2024.

**Methods:**

Choroidal OCT-A sections were analyzed using an ImageJ-based open-source software (OCTAVA).

**Main Outcome Measures:**

To assess the comparison between CM and CN using vessel area density, vessel length density, total vessel length, mean vessel diameter, and tortuosity index.

**Results:**

Thirty patients with CM and 30 patients with CN could be included in the study. Choroidal melanoma showed significantly reduced vessel area density (30.46% vs. 37.10%; *P* < 0.001), vessel length density (1.83% vs. 2.40%; *P* < 0.001), total vessel length (11.8 vs. 19.7 mm; *P* < 0.001), and tortuosity index (0.140 vs. 0.155; *P* = 0.008) compared with CN, whereas vessel diameter showed no significant difference. Receiver operating curve analysis demonstrated good diagnostic performance for vessel area density, vessel length density, and total vessel length (area under the curve: 0.81–0.86).

**Conclusions:**

OCT angiography using open-source software quantitative choroidal vasculature analysis may play a future role in objective differentiation between CM and CN. Future role of OCT-A in multimodal imaging for melanocytic choroidal tumors must, however, be further validated in larger, prospective trials and across different, preferably commercially available OCT platforms.

**Financial Disclosure(s):**

The authors have no proprietary or commercial interest in any materials discussed in this article.

Choroidal melanoma (CM) is the most common intraocular malignancy in adulthood with an annual incidence of about 6 per million in Europe and North America.^1^ Choroidal melanoma must be differentiated from choroidal nevus (CN), which is a benign choroidal tumor without need for treatment, even though a malignant transformation is possible in 1 of 8845 cases.^2^ The distinction between both entities is crucial to prevent unnecessary treatment and harm to vision, as well as prevent metastatic risk for untreated malignancies.

Differential diagnosis between these 2 entities is predominantly made clinically, using primary indirect fundoscopy and slitlamp biomicroscopy. Furthermore, multimodal imaging, such as ultrasonography, fundus photography, autofluorescence, and OCT, supports clinical interpretation and diagnosis. Their importance has been underlined by their use and adoption in clinical scores and criteria such as the Mushroom shape, Orange-Pigment, Large Size, Enlargement, Subretinal Fluid (MOLES) ^3^ or To Find Small Ocular Melanome - Doing IMaging (TFSOM-DIM) score.^4^

Nine distinct intrinsic histopathologically defined vascular patterns have been described in uveal melanomas.^5^ Some of those are associated with more aggressive uveal melanomas with high metastatic disease. In contrast, CN commonly respects the normal choroidal vasculature because nevus cells grow around the normal deeper choroidal vessels and underneath the choriocapillaris. In contrast, CM inhibits intrinsic alteration in microcirculation and vascular architecture with the described 2-dimensional, distinct vascular patterns. A true in vivo angiographic correlation to these patterns has not been unequivocally established. OCT angiography (OCT-A) is a relatively new image modality that—in comparison to fluorescein or indocyanine green angiography—has the advantages of being noninvasive and dye-free, which avoids systemic adverse effects. Retinal and choroidal microvasculature can be visualized by detecting blood flow via OCT-based laser light reflectance.^6^ OCT angiography has been widely adopted in several retinal pathologies such as diabetic retinopathy^7^ or age-related macular degeneration.^8^ Moreover, it is possible to produce 2-dimensional angiographic slabs that are theoretically comparable to histopathologic sections.

OCT angiography for differentiation between CM and CN has not yet been widely used as a diagnostic tool, even though a handful of studies have been conducted regarding this question.^9^, ^10^, ^11^, ^12^ The data published so far, however, focused mainly on descriptive distinctions such as visualization of intrinsic microcirculation networks or abnormal vasculature in CM compared with CN. Minimizing subjective reader bias, a quantitative and objective differentiation of vascular architecture would be desirable. Thus, the aim of this study was to obtain such an analysis and differentiation of CM and CN choroidal microcirculation by using an open-source software.

## Methods

We conducted a retrospective case-control study in the Department of Ophthalmology, University Hospital Essen, Germany. We included all treatment-naïve patients with CM and CN who received an OCT-A from November 2022 to December 2024. Exclusion criteria were insufficient image quality, previous treatment of the tumor, tumors anterior to the equator, CN with choroidal neovascularization, and the presence of any other ocular comorbidity that could influence choroidal vasculature (adult macular degeneration, high myopia, choroiditis/chorioretinitis). The following clinical data were collected for all patients: age, sex, tumor thickness, and maximal/minimal tumor diameter (measured either by OCT or ultrasonography), best-corrected visual acuity, presence of exudation, measure of diagnosis (for CM), location of the tumor, and grade of pigmentation (melanotic vs. amelanotic).

OCT angiography was assessed with SPECTRALIS H+OCT device (Heidelberg Engineering) using a 15° × 15° field of view. Images were reviewed using the Heidelberg HeyEx Software (version 2.5.5). To analyze the choroidal vasculature, we primarily obtained the segmentation algorithm from the software using the choroid rather than the choriocapillaris scan to obtain as much volume as possible. If segmentation was insufficient or erroneous, manual segmentation was used. A representative enface scan of the choroid in the center of the tumor—containing as much tumor vasculature as possible—was saved. Besides maximal visible choroidal vessel depth was measured manually in the B-Scan, measuring the distance between Bruch membrane and the deepest vessel signal.

**Figure 1:**
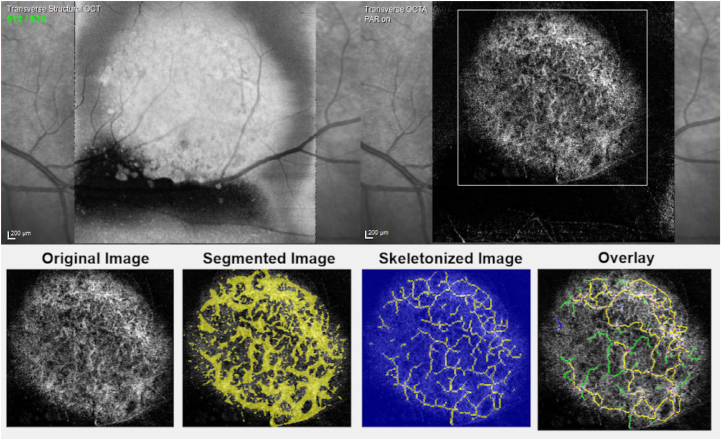
Example of vasculature analysis of a choroidal melanoma using Image-J-based open-source software OCTAVA for OCT angiography. A region of interest was chosen to obtain as much tumor vasculature as possible and to minimize inclusion of image artifacts or areas of nonvisualization, as can be seen in the upper right picture in white. For the region of interest, transversal structural OCT (in the upper left) was used as orientation in a consensus of 2 readers. After binarization and skeletonization, an overlay is generated, of which metrics can be made for vessel diameters, lengths, and tortuosity.

Vasculature analysis was done using an ImageJ-based open-source software (OCTAVA—OCTA Vascular Analyzer^13^) that was made for quantitative OCT-A vasculature analysis. The scan of each tumor was imported into OCTAVA, and a representative region of interest (ROI) for each tumor was determined for examination. The definition of ROI was depicted by 2 readers (T. Ku and T. Ki) in consensus and was assessed by using as much tumor vasculature and leaving out as much normal choroidal vasculature as possible. As such, transversal structural OCT images were used as a marker for tumor margins and for the definition of ROI. Additionally, large areas of shadowing, image artifacts, and nonvisualization of vasculature due to tumor thickness were kept out of the ROI. Images were first binarized and then skeletonized before data measurements were made by the software (Fig 1). For further examination, the following quantitative vasculature data were collected for each tumor: vessel area density (in percent), vessel length density (in percent), total vessel length (in mm), mean vessel diameter (in μm), and tortuosity index.

Statistical analysis was done using SPSS 22 (Statistical Package for Social Science, IBM). Dichotomous variables are presented as absolute and relative frequencies (n, percent) and continuous variables as mean ± standard deviation (SD). Statistical significance was asserted for *P* values <0.05, and a 3-tailed *t* test was used for comparison of means and the *χ*^2^ test for comparisons of proportions. A 2-sided Pearson correlation test was done, analyzing the correlation between maximal tumor thickness and maximal choroidal vessel depth. The receiver operating curve was analyzed for significant vasculature parameters, generating an area under the curve calculation.

We followed the Declaration of Helsinki from 1975. The study was approved by the Institutional Board of University Duisburg-Essen with the following approval number: 25-12354-BO.

## Results

Sixty patients could be included in the study: 30 with CM and 30 with CN. Clinical data of the CM and CN groups can be found in Table 1. Diagnosis in the CM group was made via (transretinal) biopsy and vitrectomy in 3 eyes, documented tumor growth in 8 eyes, and clinical diagnosis by using TFSOM-DIM criteria,^4^ if there was evidence of ≥4 risk factors, in 19 eyes. There was no difference in age and sex distribution between the 2 groups. Tumor thickness and maximal/minimal tumor diameter were higher in the CM group (2.3 mm [SD: ±1.0 mm] vs. 0.9 mm [SD: ±0.4 mm]; *P* < 0.001 and 8.0/10.1 mm [SD: ±2.6/3.8 mm] vs. 3.4/4.0 mm [SD: ±1.6/1.7 mm]; both *P* < 0.001) respectively. Best-corrected visual acuity was worse in the CM group (0.4 logarithm of the minimum angle of resolution [logMAR] [SD: ±0.4 logMAR] vs. 0.1 logMAR [SD: ±0.1 logMAR]; *P* < 0.001) with more tumors showing exudation (n = 30 [100%] vs. n = 5 [17%]; *P* < 0.001). In the CM group, tumor location was distributed in the following order: fovea, n = 13 (43.3%); juxtapapillary, n = 6 (20.0%); vascular arcades, n = 6 (20.0%); temporal to fovea, n = 4 (13.3%), and circumpapillary, n = 1 (3.3%). Locations of CN were different with the following distribution: vascular arcades, n = 13 (43.3%), juxtapapillary, n = 7 (23.3%), temporal to fovea, n = 4 (13.3%), fovea, n = 3 (10%), and peripheral, n = 3 (10%). Maximal visualized choroidal vessel depth was significantly deeper with 312 μm (SD: ±85 μm) in the CM group compared with 267 μm (SD: ±68 μm) in the CN group (*P* = 0.027). There is a moderate positive correlation between tumor thickness and maximal choroidal vessel depth (*r* = 0.389; *P* = 0.002).

**Table 1:** Clinical Data Overview of Study Population

	Choroidal Melanoma	Choroidal Nevus	
Age (y)	61 (±14)	61 (±12)	*P* = 1.000
Sex	M = 16/F = 14 (53% and 47%)	M = 15/F = 15 (both 50%)	
VA (logMAR)	0.4 (±0.4)	0.1 (±0.1)	*P* < 0.001
Max. thickness (mm)	2.3 (±1.0)	0.9 (±0.4)	*P* < 0.001
Min. diameter (mm)	8.0 (±2.6)	3.4 (±1.6)	*P* < 0.001
Max. diameter (mm)	10.1 (±3.9)	4.0 (±1.7)	*P* < 0.001
Exudation	n = 30 (100%)	n = 5 (17%)	*P* < 0.001
Tumor location			
Fovea	n = 13 (43.3%)	n = 3 (10.0%)	
Juxtapapillary	n = 6 (20.0%)	n = 7 (23.3%)	
Vascular arcades	n = 6 (20.0%)	n = 13 (43.3%)	
Temporal to fovea	n = 4 (13.3%)	n = 4 (13.3%)	
Circumpapillary	n = 1 (3.3%)	–	
Peripheral	–	n = 3 (10.0%)	

F = female; logMAR = logarithm of the minimum angle of resolution; M = male, Max.= maximal; Min.= minimal; VA = visual acuity.

Vasculature analysis (Table 2) showed statistically significant differences between CM and CN in mean vessel area density (30.46% [SD: ±6.0%] vs. 37.10% [SD: ±4.8%]; *P* < 0.001), vessel length density (1.83% [SD: ±0.41%] vs. 2.4% [SD: ±0.35%]; *P* < 0.001), total vessel length (11.8 mm [SD: ±5.4 mm] vs. 19.7 mm [SD: ±5.2 mm]; *P* < 0.001), and tortuosity index (0.140 [SD: ±0.022] vs. 0.155 [SD: ±0.020]; *P* = 0.008). There was no difference in mean vessel diameter (42.8 μm [SD: ±6.4 μm] vs. 40.0 [SD: ±7.2 μm]; *P* = 0.112). Using receiver operating curve analysis area under the curve for vessel area density, vessel length density and total vessel length showed good performance for discrimination between CM and CN considering vessel area density, vessel length density, and total vessel length (area under the curve: 0.81 [95% CI: 0.70–0.92], 0.86 [95% CI: 0.77–0.96] and 0.85 [95% CI: 0.75–0.94]). Area under the curve for tortuosity index showed moderate performance (0.71 [95% CI: 0.58–0.84]).

**Table 2:** Choroidal Vasculature Analysis Compared between Choroidal Melanoma and Nevi

	Choroidal Melanoma	Choroidal Nevus	
Max. vessel depth (μm)	312 (±85)	267 (±68)	*P* = 0.027
Vessel area density (%)	30.5 (±6.0)	37.1 (±4.8)	*P* < 0.001
Vessel length density (%)	1.83 (±0.41)	2.40 (±0.35)	*P* < 0.001
Total vessel length (mm)	11.8 (±5.3)	19.7 (±5.2)	*P* < 0.001
Mean diameter (μm)	42.8 (±6.4)	40.0 (±7.2)	*P* = 0.112
Tortuosity index	0.140 (±0.02)	0.155 (±0.02)	*P* = 0.008

Max. = maximal.

No statistically significant difference between CM groups was diagnosed clinically using risk factors versus documented growth and biopsy (n = 19 vs. 11) regarding vasculature analysis: vessel area density (32.5% [SD: ±5%] vs. 30.5 [SD: ±7%]; *P* = 0.994), vessel length density (1.8% [SD: ±0.5%] vs. 1.8% [SD: ±0.5%]; *P* = 0.960), total vessel length (11.4 mm [SD: ±5 mm] vs. 12.5 mm [SD: ±7 mm]; *P* = 0.639), mean diameter (44.3 μm [SD: ± 6 μm] vs. 40.2 μm [SD: ±6 μm]; *P* = 0.102), and tortuosity (0.14 [SD: ±0.02] vs. 0.14 [SD: ±0.03]; *P* = 0.594).

If compared between amelanotic and melanotic CM (n = 7 vs. 23), there was no statistic difference between 2 groups in any of the mentioned parameters: vessel area density (32.1% [SD: ±5%] vs. 30.0 [SD: ±6%]; *P* = 0.360), vessel length density (1.9% [SD: ±0.3%] vs. 1.8% [SD: ±0.4%]; *P* = 0.417), total vessel length (13.2 mm [SD: ±6 mm] vs. 11.3 mm [SD: ±5 mm]; *P* = 0.435), mean diameter (45.7 μm [SD: ±7 μm] vs. 41.9 μm [SD: ±6 μm]; *P* = 0.242), and tortuosity (0.14 [SD: ±0.02] vs. 0.14 [SD: ±0.02]; *P* = 0.955). Likewise, this was true if comparing all amelanotic CN + CM versus melanotic CN + CM (n = 13 vs. 47): vessel area density (34.1% [SD: ±5%] vs. 33.7 [SD: ±7%]; *P* = 0.826), vessel length density (2.1% [SD: ±0.4%] vs. 2.1% [SD: ±0.5%]; *P* = 0.814), total vessel length (16.2 mm [SD: ±6 mm] vs. 15.6 mm [SD: ±7 mm]; *P* = 0.746), mean diameter (41.9 μm [SD: ±9 μm] vs. 41.2 μm [SD: ±6 μm]; *P* = 0.798), and tortuosity (0.14 [SD: ±0.01] vs. 0.15 [SD: ±0.02]; *P* = 0.142).

For further differentiation, the CN group was separated into suspect nevi (sCN) and common nevi. Suspected nevi were defined as CN with ≥1 risk factors using the TFSOM-DIM criteria^4^ that were not diagnosed as CM. Using this differentiation, 12 tumors were assessed as sCN (40%) and 18 tumors as common nevi (60%). Vasculature analysis between these 2 groups differed significantly only in total vessel length (17.1 mm [SD: ±5.1 mm] vs. 21.4 mm [SD: ±4.5 mm]; *P* = 0.0217). All other vascular analysis parameters showed no statistically significant differences between these 2 groups.

Differentiation between CM and sCN showed significant differences between both groups for vessel area density (30.46% [SD: ±6.0%] vs. 36.00% [SD: ±3.1%]; *P* < 0.01), vessel length density (1.83% [SD: ±0.41%] vs. 2.29% [SD: ±0.30%]; *P* < 0.001), and total vessel length (11.8 mm [SD: ±5.4 mm] vs. 17.1 mm [SD: ±5.4 mm]; *P* = 0.009).

## Discussion

**Figure 2:**
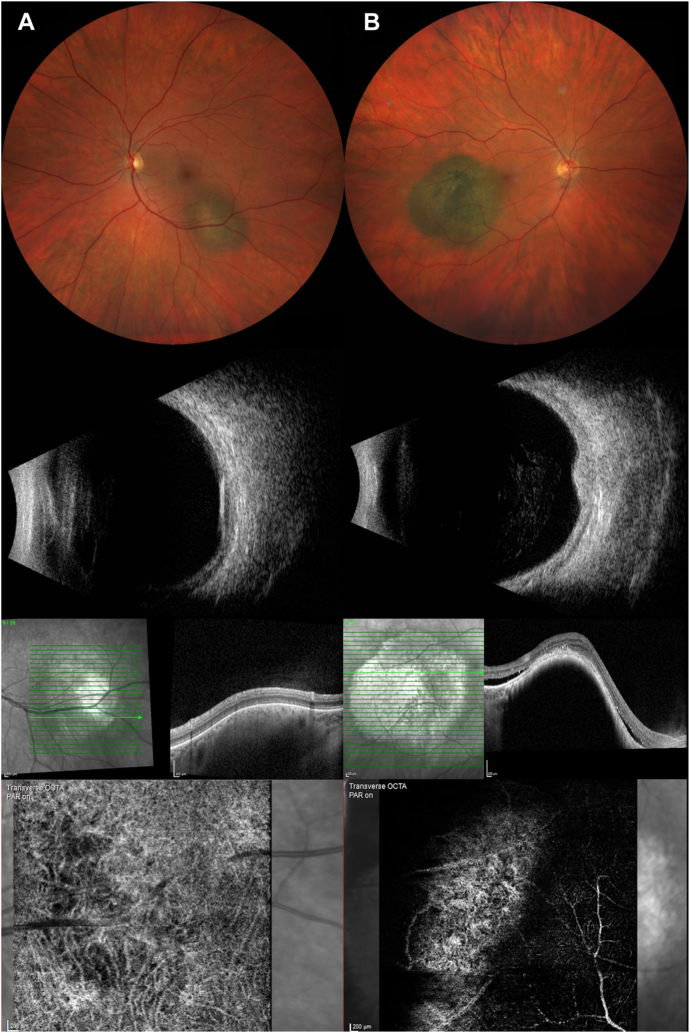
Example of a choroidal nevus (**A**) and melanoma (**B**). (**A**) Choroidal nevus shows no clinical sign of orange pigment, a maximal diameter of 4.9 mm, a thickness of 1.3 mm in ultrasonography (longitudinal 5 o’clock), no signs of subretinal fluid in OCT, and a normal choroidal vasculature in OCT angiography with a vessel area density of 39% and total vessel length of 14.9 mm. (**B**) Choroidal melanoma with clinical orange pigment, a maximal diameter of 10.2 mm, a thickness of 2.1 mm in ultrasonography (longitudinal 8 o’clock), subretinal fluid in OCT, and changed choroidal microcirculation with a vessel area density of 27% and a total vessel length of 8.9 mm.

In this study, we demonstrated that differentiation in choroidal vasculature between CM and CN can be objectively distinguished using OCTAVA, an open-source software. Choroidal melanoma vasculature analysis showed a significant reduction of vessel area density, vessel length density, and total vessel length compared with CN. Furthermore, sCN showed alterations in choroidal vasculature compared with CM (Fig 2). Tortuosity was significantly reduced in the CM group compared with CN, whereas there was no difference in mean tumor diameter between the 2 groups. Furthermore, microcirculation differences could be measured between sCN and CN and sCN and CM, on the other hand.

**Figures 3:**
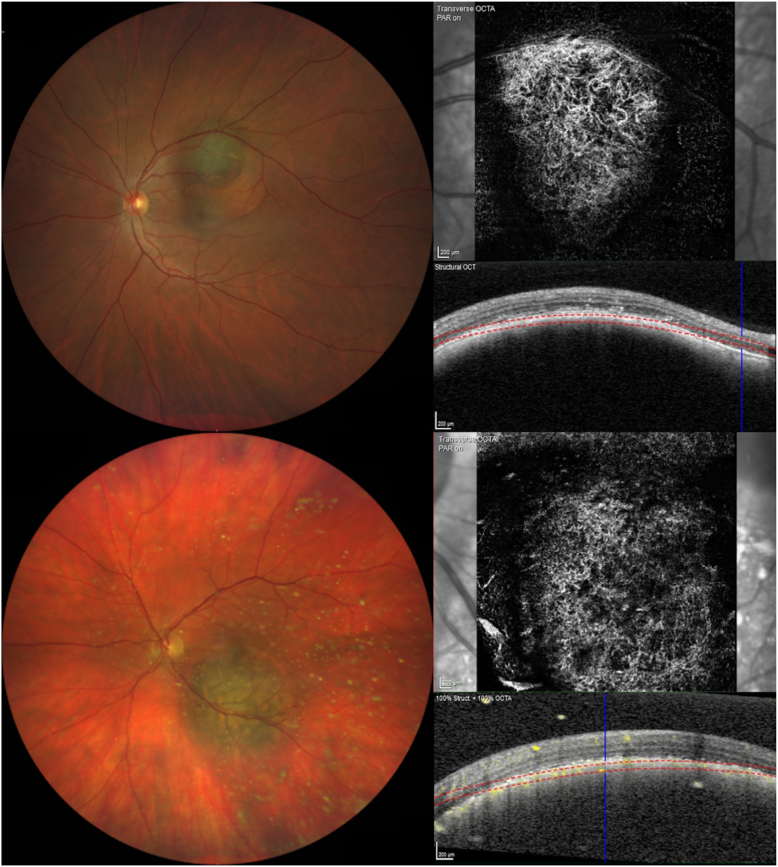
Two examples of choroidal vascular abnormalities in choroidal melanoma using OCT angiography.

These objectively measurable differences between the 2 entities reflect choroidal microcirculation alterations in CM, which can be visualized with OCT-A (Fig 3). Angiogenesis in tumors not only results in the formation of new blood vessels but also in vasculature remodeling. Microcirculation changes have been demonstrated in CM histopathologically, demonstrating different microcirculation patterns,^14^ which can even possibly predict a patient’s prognosis.^15^ Histopathologic differentiation in microcirculation architecture between CM and CN has been demonstrated before, showing a spectrum of benign normal choroidal vasculature over rather benign microcirculation alteration patterns to changes such as network and loops in CM with worse prognosis.^5^ This spectrum or possibly transformation process can hypothetically be visualized with OCT-A because some nevi tend to develop intrinsic microcirculation, which is emphasized by the difference in vascular analysis between normal and suspicious nevi. On the other hand, the higher vascular density found in CN could represent the high vascular density of the intact choriocapillaris, which is not present in CM, which infiltrate and destroy adjacent normal vascular structures. Based on these observations, we tried to differentiate between sCN and common CN to analyze possible measurable vasculature alterations, which showed a significant reduction of vessel length density in sCN.

Compared with fluorescein and indocyanine green angiography, which are invasive and dye-dependent techniques, OCT-A has the capability to generate images of retinal and choroidal vasculature in a fast manner, avoiding possible side effects. So far, a few studies have investigated the use of OCT-A for the purpose of differentiation between malignant and benign melanocytic choroidal tumors.^9^, ^10^, ^11^, ^12^ These studies demonstrate the possibility of differentiation between CM and CN, showing microcirculation changes in the melanoma group. However, these studies focused mainly on descriptive visualization of microcirculation alterations and patterns, distinguishing between the 2 entities. Visualization, imaging, and digital analysis of vasculature alterations can add an objective, quantifiable parameter for differentiation and comparison of microcirculation changes, which are observer-independent and have not been demonstrated in the literature previously. Greig et al^12^ demonstrated significant differences in choroidal vessel depth, the deepest choroidal vessel visualized, and the presence of deep choroidal vessels between CM and CN in a small patient cohort. These findings could be explained by tumor thickness differences because moderate but significant positive correlation between maximal tumor thickness and maximal choroidal vessel depth was observed in our population. To address possible confounding factors regarding tumor pigmentation—which possibly can affect vascular analysis—we compared amelanotic versus melanotic in the CM and overall cohort and showed no difference between the groups. However, groups in this way were not equally balanced, so this finding has to be interpreted with caution, and vasculature analysis addressing tumor pigmentation should be further investigated in the future.

Our results display several advantages compared with previously published studies. First, we could objectively measure choroidal microcirculation alterations and found significant differences between melanoma and nevi, which are as independent as possible of a possible observer bias. For this purpose, open-source software was used, which is independent of the used OCT-A hardware and would be comparable between different OCT-A devices, even though this was beyond the scope of this study. Moreover, we examined a representative patient cohort that could demonstrate a significant distinction between CM and CN in choroidal microcirculation architecture. Nevertheless, these findings must be validated in different and larger patient cohorts. A disadvantage of our study is the lack of histopathologic or molecular genetic confirmation of the clinical diagnosis in most cases, with only 3 tumors that received biopsy, and the majority of CM were diagnosed clinically using multimodal imaging only. To address this, we compared different CM groups and showed significant differences in parameters regardless of the diagnosis approach.

The use of OCT-A in ocular oncology is limited by notable drawbacks that must be addressed and have been evident in our study. The current OCT devices have a limited visualization of deeper choroidal vasculature structures other than choriocapillaris and superficial Sattler layer, which is highlighted by the maximal depth of vessel visualization in our cohort. However, compared with a study using swept-source OCT-A for melanoma and nevi with a deepest choroidal vessel visualization of 180 μm, we were able to scan as deep as 312 μm for CM.^12^ Nevertheless, this imaging penetration limits the ability to analyze thicker choroidal tumors and their deeper intrinsic vasculature. Even though, depth of visualization can be possibly addressed with future developments, we believe that this can be negligible for our study because it was our aim to distinguish between small CM and CN, which is clinically more challenging. Thicker CMs are more easily differentiated in clinical practice. One rationale for using an open-source software for the purpose of this study is the fact that objective measurements of retinal and choroidal vasculature are not feasible with all commercial OCT devices and, for instance, were not usable for the device used in this study. Because this open-source software is possible device-independent, its utility in clinical practice is questionable, and limits its daily use in clinical practice. It is worth mentioning that acceptable image quality could be gained only for rather central tumors in this and other studies; however, we are sure these problems can be solved by the development and advances of widefield OCT-A devices.^16^ Furthermore, it must not be neglected that OCT-A is accompanied by imaging artifacts in prominent lesions, which makes sufficient vasculature presentation and analysis impossible. This is why tumors with insufficient image quality were excluded in this study, and tumors in the CM had a comparably small maximal tumor thickness with 2.3 mm (SD: ±1 mm) as stated earlier, which limits the current ability to use this technique for a wide range of patients with CM. On the other hand, tumor thickness was still statistically significantly lower in the CN group. Therefore, it must be kept in mind that vasculature difference could possibly also be explained by additional artifacts induced by higher tumor thickness in the CM group. It is to noting that the depiction of ROI with this software and in general is not free of reader-dependent bias, and careful assessment needs to be done to analyze tumor vasculature only free of nontumor vessels or image artifacts of any kind. To achieve the best results definition of ROI was done by 2 readers in consensus, and only OCT-A images with sufficient image quality were included in this study.

In conclusion, we could demonstrate that an objective additional distinction between CM and CN is possible by analyzing choroidal vasculature with OCT-A using an open-source software. Our study suggests that OCT-A could be included next to the ultrasound, autofluorescence, OCT, and fluorescein angiography/indocyanine green angiography in the multimodal imaging for the differentiation between CM and CN. Further research in different and larger patient cohorts is necessary to validate these findings. Additionally, confirmation and comparison of the presented findings with different OCT devices would be desirable, possibly using commercially available solutions that could easily be integrated into clinical practice.
